# Low incidence of the immune reconstitution inflammatory syndrome among HIV-infected patients starting antiretroviral therapy in Gabon: a prospective cohort study

**DOI:** 10.1007/s15010-017-1000-9

**Published:** 2017-03-27

**Authors:** S. Janssen, K. Osbak, R. Holman, S. Hermans, A. Moekotte, M. Knap, E. Rossatanga, M. Massinga-Loembe, A. Alabi, A. Adegnika, C. Meenken, M. van Vugt, P. G. Kremsner, G. Meintjes, T. van der Poll, M. P. Grobusch

**Affiliations:** 1 Division of Internal Medicine, Department of Infectious Diseases, Center of Tropical Medicine and Travel Medicine, Academic Medical Center, University of Amsterdam, Meibergdreef 9, 1100 DD Amsterdam, The Netherlands; 2 Centre de Recherches Médicales en Lambaréné, Lambaréné, Gabon; 3 Centre de Traitement Ambulatoire Lambaréné, Lambaréné, Gabon; 4 Institute of Tropical Medicine, University of Tübingen, Tübingen, Germany; 5 Clinical Infectious Diseases Research Initiative, Institute of Infectious Disease and Molecular Medicine, University of Cape Town, Cape Town, South Africa; 6 Division of Internal Medicine, Department of Infectious Diseases, Center for Experimental and Molecular Medicine, Academic Medical Center, University of Amsterdam, Amsterdam, Netherlands; 7 Clinical Research Unit, Academic Medical Center, University of Amsterdam, Amsterdam, The Netherlands; 8 Department of Global Health, Academic Medical Center, Amsterdam Institute for Global Health and Development, University of Amsterdam, Amsterdam, The Netherlands; 9 Faculty of Health Sciences, Desmond Tutu HIV Centre, Institute for Infectious Disease and Molecular Medicine, University of Cape Town, Cape Town, South Africa; 10 Department of Internal Medicine, School of Medicine, Makerere University College of Health Sciences, Kampala, Uganda; 11 Department of Ophthalmology, VU Medical Center, Vrije Universiteit, Amsterdam, The Netherlands

**Keywords:** HIV, AIDS, Tuberculosis, Opportunistic infections

## Abstract

There is a paucity of data on the immune reconstitution inflammatory syndrome (IRIS) in the Central African region. We followed ART-naive HIV-infected patients initiating antiretroviral therapy in an HIV clinic in Gabon, for 6 months. Among 101 patients, IRIS was diagnosed in five. All IRIS cases were mucocutaneous manifestations. There were no cases of tuberculosis (TB) IRIS, but active TB (*n* = 20) was associated with developing other forms of IRIS (*p* = 0.02). Six patients died. The incidence of IRIS is low in Gabon, with mild, mucocutaneous manifestations.

**Electronic supplementary material:**

The online version of this article (doi:10.1007/s15010-017-1000-9) contains supplementary material, which is available to authorized users.

## Introduction

Immune reconstitution inflammatory syndrome (IRIS) is one of the complications of antiretroviral therapy (ART) leading to morbidity in HIV-infected patients [1]. IRIS can occur with any opportunistic infection, and features two distinct forms: paradoxical IRIS, with paradoxical worsening of a known pre-existing infection; or unmasking IRIS, when a previously undiagnosed condition in HIV-infected patients is revealed with exaggerated local or systemic inflammation, soon after the commencement of ART [1].

Although many studies have reported on IRIS incidence in a variety of epidemiologic settings [1], there is a paucity of published data on the incidence and risk factors of the syndrome in the Central African region [2]. Characteristics of this setting differ from other regions of the world in several aspects, including a high burden of co-existing parasitic infections [3], a high genetic variability of HIV [4] and the equatorial latitude contributing to a decreased likelihood of vitamin D deficiency [5].

Despite the decrease of HIV-associated mortality since the implementation of ART, mortality remains high in certain patient groups, especially early after starting ART [6]. Tuberculosis (TB) is an important cause of early mortality after ART initiation [6], even when patients receive treatment for both infections in line with international guidelines. The causes of this high mortality remain to be elucidated.

In this prospective study, we describe the incidence and risk factors for IRIS in Lambaréné, Gabon. Secondly, we determine mortality during the first 6 months after ART initiation and investigate associated risk factors.

## Materials and methods

Recruitment for this prospective cohort study was performed in Lambaréné, Gabon. The HIV seroprevalence is estimated at around 4.1% in Gabon [7]. Lambaréné is a town of 35,000 inhabitants situated within the Central African rainforest area of the Moyen Ogooué province. Patients were recruited at the regional HIV clinic (Centre de Traitement Ambulatoire). ART was initiated if patients had CD4 counts less than 350 cells/µL or were symptomatic [World Health Organization (WHO) stage 3 or 4] [8], in accordance with the Gabonese national guidelines. In patients co-infected with TB, ART was initiated after the two-month intensive phase of TB treatment, or 2 weeks after TB treatment initiation, if CD4 counts were less than 100 cells/µL.

Ethical clearance was obtained from the Institutional Review Board of the Centre de Recherches Médicales de Lambaréné (CERMEL). All participants provided written informed consent.

**Fig. 1:**
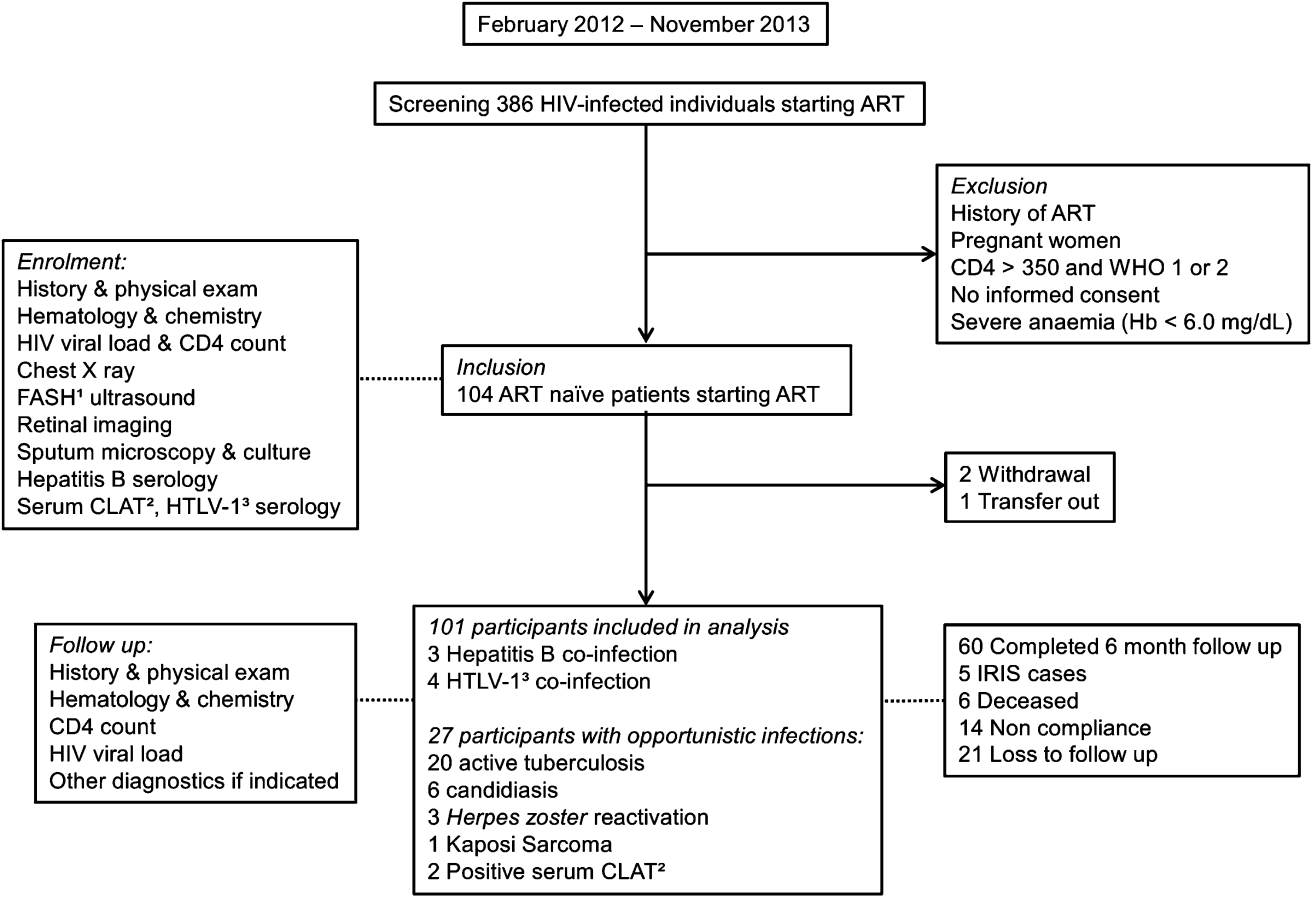
Study flow. Figure showing the study flow from screening to enrolment and follow-up. Twenty-seven patients were diagnosed with one or more opportunistic infections at enrolment. ^1^Focused Assessment with Sonography for HIV-associated TB (FASH), ^2^Serum cryptococcal latex antigen test (serum CLAT), ^3^human T-cell lymphotropic virus 1 (HTLV-1)

ART-naïve patients were followed for 6 months after they initiated ART. From February 2012 to November 2013, all adult (≥18 years old) ART-naïve patients starting ART at the clinic were screened for eligibility. Exclusion criteria were pregnancy, history of ART and severe anemia (hemoglobin <6.0 g/dL, in view of repeated blood draws) (Fig. 1). Follow-up visits were performed at one, three and six months after patients started ART, with additional visits in case of new health complaints.

The estimated sample size was 100 patients, among which we expected to find 30 IRIS cases, based on previous research from epidemiologically comparable settings [1]. As recruitment was challenging and slower than expected, the recruitment interval was prolonged from 12 to 21 months.

Basic demographic data, World Health Organization staging [8] and medical history including previous illnesses and opportunistic infections were obtained at enrolment. An extensive physical exam was performed by one of the study physicians (SJ or KO), including a basic neurologic exam, visual acuity testing and a full inspection of the skin for cutaneous abnormalities. At follow-up visits, information on any new health complaints and self-reported ART compliance was documented and the physical exam was repeated.

At enrolment and follow-up visits, full blood counts and basic chemistry tests were done at the CERMEL laboratory. Baseline hepatitis B serology HIV viral loads were measured, using the COBAS TaqMan 48 system (Roche Molecular Systems Inc., Pleasanton, CA, USA). CD4 counts were acquired using a *BD FACSCount™* system (Becton–Dickinson, Franklin Lakes, NJ, USA). CD4 counts and HIV viral loads were measured at baseline, month 3 and month 6, and at the time of an IRIS event. Samples were transported to the Academic Medical Center, where the serum cryptococcal latex antigen test (CLAT) and serology for human T-cell lymphotropic virus 1 (HTLV-1) were performed on enrolment serum samples.

All patients were evaluated for signs and symptoms of TB (cough, fever, night sweats and possible close contact with TB infected individuals). Baseline chest radiography was performed, and a Focused Assessment with Sonography for HIV-associated TB (FASH), to increase diagnostic yield of the screening process [9]. FASH was performed in the context of an ongoing study and was not part of routine clinical care in Gabon. Sputum collection was attempted in all patients. Where possible, one spot sample and 2 morning sputa were sent for fluorescent microscopy using auramine-rhodamine staining performed at CERMEL. Samples were sent for culture applying a previously described procedure [10]. In the instance of any TB symptoms at follow-up visits, diagnostic procedures were repeated.

To diagnose ophthalmological manifestations of IRIS, we performed baseline retinal imaging and in case of a deterioration in visual acuity at follow-up visits using a PanOptic ophthalmoscope (Welch-Allyn, Skaneateles Falls, NY, USA). Images were captured on an iPhone 4 (Apple Inc., Cupertino, CA, USA) and sent for inspection by an ophthalmologist specialized in HIV-related disorders at the VU Medical Center in the Netherlands (CM).

Enrolment and follow-up data were captured in an OpenClinica^®^ database (Waltham, MA, USA).

Main outcomes for this study were development of IRIS and 6-month mortality. We used criteria of the International Network for the Study of HIV-associated IRIS (INSHI) [11, 12] for defining IRIS events. In brief, paradoxical IRIS was defined as a clinical worsening of a known and adequately treated pre-existing infection with response to ART, in the absence of drug resistance or side effects of drugs used; unmasking IRIS was defined as an exaggerated local or systemic inflammation as a result of an undiagnosed condition, soon after the commencement of ART. Each suspected case was discussed by three investigators (SJ, KO and MPG). Information on mortality was supplemented using the HIV clinic’s register for deceased patients.

In case of a delayed clinic visit, multiple phone calls were made to urge the patient to attend the clinic. Loss to follow-up was defined as a patient delaying clinic visits of more than 2 weeks. Non-compliance was defined as a self-reported interruption of ART for at least 2 weeks.

All clinical visits were performed by one of the two study physicians (SJ and KO); patients were seen by the same physician at subsequent visits in most cases to reduce inter-observer bias. Patients were supported with a reimbursement of transport costs to reduce barriers to coming to the clinic outside of scheduled visits in case of deterioration.

We compared patients by IRIS and survival status. To evaluate selection bias, we compared patients included in the primary analysis with those, who became LTFU or were excluded due to non-compliance. Data were assessed for completeness. If for a certain factor >10% of data were missing, patient characteristics for the group with missing data were compared to those with complete data.

Patient groups were compared using Fisher’s exact test for categorical data and Mann–Whitney U test for continuous data. Kaplan–Meier curves were used to calculate the cumulative incidence of IRIS and six month mortality using percentages and 95% confidence intervals. Log-rank tests were applied to examine associations between baseline characteristics and time to IRIS event or mortality. Patients LTFU or excluded because of non-compliance were censored. We used SPSS Statistics Version 21 (IBM, Chicago, IL, USA) and GraphPad PRISM Version 6 (San Diego, CA, USA).

## Results

From February 2012 to November 2013, 386 HIV-infected patients were screened; 104 individuals were recruited, of which 101 were included in the final analysis (Fig. 1). Twenty-seven patients were diagnosed with one or more opportunistic infections at enrolment; 20 patients were diagnosed with active TB; 13/20 had pulmonary TB, 7/20 had extrapulmonary manifestations. TB was microbiologically confirmed in 6/20 patients. All of them started TB treatment before ART initiation. Furthermore, one patient was diagnosed with Kaposi sarcoma, three patients had oral candidiasis, two were diagnosed with *Herpes zoster* reactivation, one had both oral candidiasis and *Herpes zoster* reactivation, and two had oral candidiasis and pulmonary TB (Fig. 1). Eighty-five patients initiated ART within one week of enrolment. The remaining 16 patients started ART after a median of 26 days (interquartile range (IQR) 9–42 days), due to diagnosis of opportunistic infections at study inclusion, or non-compliance.

Baseline characteristics of the study population are shown in the Table 1. Most participants were female, on average 38 years of age. Most patients presented in an asymptomatic stage; although 44/101 (43.5%) presented with WHO stage 3 or 4. The median CD4 count at presentation was 180 (IQR 69-252) cells/uL. Eosinophilia (>0.45 × 10^9^ cells/L) was common (18/65 (28.6%) of patients at baseline).

**Table 1:** Patient characteristics at baseline and follow-up

	Data (*n* = 101)	Total cohort (*n* = 101)	IRIS cases (*n* = 5)	*p* value^1^	Deceased (*n* = 6)	*p* value^2^
Male sex (*N*, %)	101	34 (33.7)	1 (20.0)	0.66	1 (16.7)	0.66
Age (median, IQR)	101	38 (31–47)	41 (36–48)	0.37	47 (34–57)	0.15
BMI (kg/m^2^)	96	20.9 (18.6–23.4)	18.7 (18.2–22.6)	0.31	19.7 (16.4–21.3)	0.17
WHO stage (*n*, %)	101			0.02		0.08
1		39 (38.6)	0 (0)		1 (16.7)	
2		18 (17.8)	0 (0)		0 (0)	
3		27 (26.7)	3 (60.0)		3 (50)	
4		17 (16.8)	2 (40.0)		2 (33.3)	
Baseline co-infections	101					
TB	101	20 (19.8)	3 (60)	0.05	3 (50)	0.09
Herpes zoster	101	3 (3.0)	0 (0)	0.81	0 (0)	0.45
Kaposi sarcoma	101	1 (1.0)	0 (0)	0.81	0 (0)	0.45
Candidiasis or dermatomycosis	101	6 (5.9)	1 (20.0)	0.81	0 (0)	0.45
Hepatitis B	97	3 (3.1)	0 (0)	0.85	0 (0)	0.82
Cryptococcosis	93	2 (2.2)	0 (0)	0.90	0 (0)	0.89
HTLV-1	101	4 (4.0)	0 (0)	0.81	0 (0)	0.78
Laboratory diagnostics						
Hemoglobin (g/dL)	97	10.1 (8.6–12.2)	8.3 (7.1–10.0)	0.06	6.2 (5.6–9.3)	0.008
White cell count (*10e9/L)	96	4.1 (3.3–5.3)	4.1 (3.8–6.0)	0.47	5.3 (2.9–6.2)	0.49
Neutrophils (*10e9/L)	71	1.73 (1.26–2.38)	2.02 (1.30–2.38)	0.72	1.78 (1.11–3.73)	0.85
Lymphocytes (*10e9/L)	76	1.29 (0.97–1.77)	1.12 (0.81–2.65)	0.95	1.46 (0.64–2.17)	0.75
Monocytes (*10e9/L)	76	0.48 (0.37–0.67)	0.72 (0.42–0.99)	0.30	0.62 (0.47–1.10)	0.20
Eosinophils (*10e9/L)	83	0.19 (0.08–0.50)	0.04 (0.02–0.07)	0.009	0.20 (0.09–0.43)	0.82
Eosinophilia (*n*, %)	65	18 (28.6)	0 (0)	0.55	1 (25)	0.68
Platelets (*10e9/L)	96	216 (159–289)	296 (199–318)	0.16	191 (145–233)	0.43
HIV viral load (log copies/mL)	84	4.87 (4.37–5.34)	5.16 (3.19–5.69)	0.68	5.43 (4.97–5.76)	0.06
CD4 count (cells/µL)	101	180 (69–252)	26 (13–170)	0.04	165 (108–237)	0.97
CD8 count (cells/µL)	92	768 (514–1227)	580 (403–1000)	0.31	597 (352–1525)	0.52
Creatinine (mmol/L)	96	59 (45–72)	64 (44–91)	0.61	52 (44–108)	1.00
Immunologic response						
Total CD4 increase during study period (cells/µL)	64	155 (49–262)	230 (78–303)	0.45	ND	ND
Viral load undetectable month 3 (*n*, %)	35	27 (77.1)	2 (50)	0.77	ND	ND
Viral load undetectable month 6 (*n*, %)	26	19 (73.1)	ND	ND	ND	ND

Patient characteristics for the total cohort; patients who developed IRIS and patients who deceased during the study follow-up

*BMI* Body mass index, *WHO* World Health Organization, *TB* Tuberculosis, *HTLV*-*1* Human T-cell lymphotropic virus-1

^1^Patients who developed IRIS versus those who did not

^2^Patients who deceased within the study period versus those who survived

Of 101 included patients, 14 were excluded due to non-compliance; 21 were LTFU and six died. Sixty patients completed the six month follow-up period. Eight patients (8/101, 7.9%) developed a suspected IRIS event. After reviewing clinical information, one case was defined as ART-associated TB rather than IRIS as there was no exaggerated inflammation, and two were defined as non-IRIS events due to a lack of evidence of immune reconstitution, as defined as lack of CD4 count increase and HIV viral load decline at the time of the event. IRIS was diagnosed in five cases (5/101; 5.0%), leading to a cumulative incidence of 6% (95% CI 0.7–12). Four patients had unmasking mucocutaneous manifestations of IRIS with new KS lesions in one patient, molluscum contagiosum in two patients and dermatomycosis in the last (prevalence unmasking mucocutaneous IRIS 4/92 patients without signs of mucocutaneous infections at baseline; 4%). Of nine patients presenting with mucocutaneous infection at baseline, one patient developed a paradoxical worsening of an ophthalmic manifestation, with worsening of skin eruptions and a decrease in visual acuity (prevalence paradoxical mucocutaneous IRIS 1/9; 11%). None of the patients diagnosed with active TB at baseline developed paradoxical TB-IRIS, and there were no unmasking TB-IRIS cases. The five IRIS events developed at a median of 44 (range 19–113) days after initiation of ART, and resolved with supportive care and continuation of ART. None of the patients died as a consequence of IRIS. Treatment with steroids or hospitalization was not required.

**Fig. 2:**
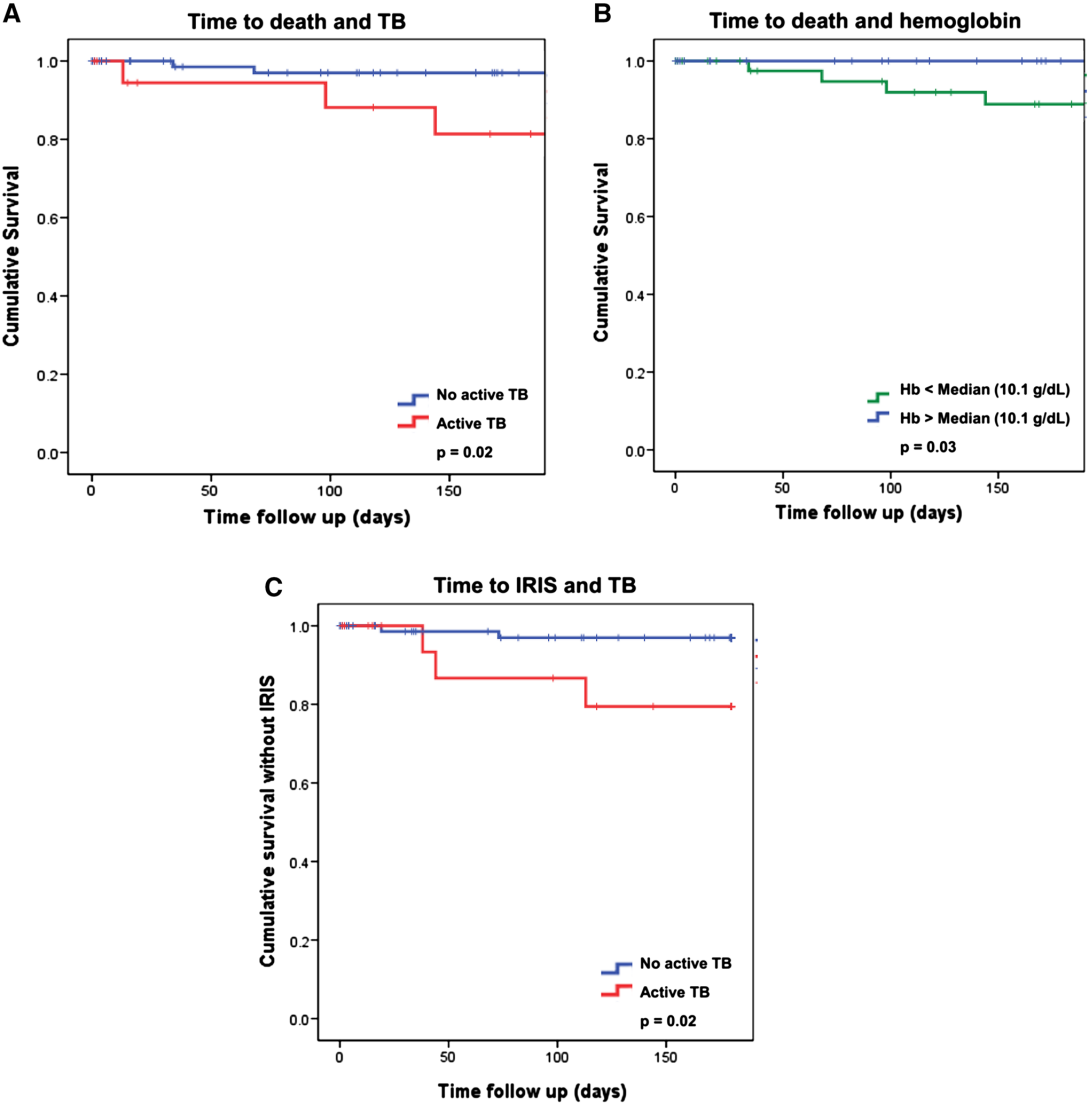
Time to death or IRIS, TB status and baseline hemoglobin. **a** is showing the cumulative survival after initiation of ART in patients with (*red line*) or without (*blue line*) diagnosis of active TB at enrolment. Diagnosis of active TB at enrolment was associated with mortality (*p* = 0.02). **b** is showing the cumulative survival after initiation of ART in patients with hemoglobin concentrations above (*blue line*) or below (*green line*) the median concentration of 10.1 g/dL. Lower hemoglobin concentrations were associated with mortality (*p* = 0.03). **c** showing the cumulative survival without IRIS after initiation of ART in patients with (*red line*) or without (*blue line*) diagnosis of active TB at enrolment. Diagnosis of active TB at enrolment was associated with development of IRIS (*p* = 0.02)

Patients who developed IRIS presented more often with a symptomatic WHO stage compared to patients who did not, had lower baseline CD4 counts, lower eosinophil counts and were more often diagnosed with active TB at baseline (Table 1). Only diagnosis of active TB at enrolment was associated with developing IRIS (*p* = 0.02) (Fig. 2).

Six patients (6/101, 6%; cumulative incidence 8%, 95% CI 7–8) died after a median of 68 (range 13–144) days. TB was diagnosed in three of them at enrolment; no additional information on the cause of death was available. Patients who died had lower hemoglobin concentrations compared to those who survived (Table 1). Lower concentrations of hemoglobin (*p* = 0.03) and diagnosis of active TB at enrolment (*p* = 0.02) were associated with time to mortality (Fig. 2a, b).

To assess selection bias, we compared baseline demographics and clinical data for patients who completed the 6-month follow-up period to those data for patients who were either LTFU or excluded because of non-compliance (Supplementary Table). There were three HTLV1 confected patients who were all in the non-compliant group. No further differences were detected. For the variables HIV viral load and eosinophil counts, there were more than 10% of data missing (Table 1). To assess for bias related to missing data, baseline demographics and clinical data for patients with missing values were compared to those with complete values. No significant differences were found (data not shown).

## Discussion

This study reports on the occurrence of IRIS and early mortality after starting ART in a primary HIV care setting in Gabon. Main findings were the low incidence of IRIS and the relatively mild manifestations of the syndrome. On the contrary, mortality during the first 6 months after ART initiation was moderately high.

The cumulative incidence of IRIS was 6%, which is relatively low compared to other low and middle income settings with a high burden of HIV and TB [1, 13]. Most cases where unmasking mucocutaneous manifestations, which were relatively mild and resolved with topical treatment and continuation of ART. Although we do report one case of ART-associated TB, no unmasking or paradoxical TB IRIS cases were seen. Our data on the incidence of unmasking TB IRIS are in line with previous reports from equatorial settings [14, 15].

Diagnosis of active TB at baseline was associated with the development of IRIS. Although no cases of TB IRIS are reported; in this case, TB might be an indicator of advanced HIV at baseline, and thus a higher risk to develop IRIS. Patients who developed IRIS presented with lower nadir CD4 counts. Both risk factors have been reported before [1, 13], supporting the generalizability of our findings.

We report a moderately high mortality in the first six months after starting ART, with TB being the major risk factor. High early mortality after initiation of ART, particularly in TB patients, is an emerging challenge in many settings, and recently this topic was placed high on the global research agenda [16].

There are several explanations for the low incidence of IRIS in this equatorial setting. Our patients underwent a thorough work-up for opportunistic infections before ART initiation, including a FASH ultrasound, potentially preventing unmasking cases. Our patients presented on average with higher nadir CD4 counts compared to cohorts from certain other settings, like Mozambique, South Africa and Uganda [15, 17, 18]. However, there is a tendency towards a lower reported incidence of unmasking IRIS in equatorial settings [13], maybe due to a lack of access to diagnostics for opportunistic infections, or due to a biological phenomenon, like a low prevalence of vitamin D deficiency or a high rate of helminthic co-infections.

Our study is limited by the high rate of LTFU and non-compliance to ART. This finding has been reported in this particular setting before [19], and is a major threat to the functioning of HIV programs in Africa. Cases of IRIS might have been missed, although this risk was reduced by facilitating presentation to the clinic by refunding travel fees. Multivariate regression analysis of factors associated with development of IRIS or mortality was not possible, due to a low number of events. According to prevalences found in previous studies [1], we expected to detect at least 30 cases of IRIS, which would have allowed for this kind of statistical analysis. Our study is underpowered to draw reliable conclusions on the occurrence of paradoxical TB and cryptococcal IRIS. No reliable data were available on the cause of death of our patients. These patients died at home or at other hospitals; none of them died at the HIV clinic. Therefore, in this setting with low resources and poor infrastructure, it was not possible to obtain reliable information on death causality.

Important strengths of our study are the prospective design and the thorough work-up of our patients to facilitate diagnosis of opportunistic infections and manifestations of IRIS. Baseline and follow-up visits were done by the same physician in most instances in order to minimize inter-observer bias and the misrecognition of IRIS events. All suspected cases were discussed by the study team to verify consistency with international definitions [11, 12].

This study represents pioneering work in this setting as there is very limited information published on the HIV and TB epidemic in Gabon [10, 19–21]. The occurrence of drug-resistant TB in this setting has only recently been recognized [10], and limited information has been reported on treatment outcomes in HIV or TB cohorts.

In conclusion, we report a low incidence of IRIS in Gabon with relatively mild manifestations. Further research on IRIS in equatorial settings is needed, to confirm this low incidence and to elucidate potential causative factors that may inform strategies to prevent the development of IRIS in high prevalence settings.

## Electronic supplementary material

Below is the link to the electronic supplementary material.

Supplementary material 1 (DOC 61 kb)
