# Supplementary material for: Low incidence of the immune reconstitution inflammatory syndrome among HIV-infected patients starting antiretroviral therapy in Gabon: a prospective cohort study
Source: Infection. 2017 Mar 27;45(5):669–76. doi: 10.1007/s15010-017-1000-9 (PMC5630650; doi:10.1007/s15010-017-1000-9)
Supplement: Supplementary file 1 — Supplementary material 1 (DOC 61 kb) [file 15010_2017_1000_MOESM1_ESM.doc]

| **Supplementary Table Patient characteristics, loss to follow up and non-compliance** | | | | | | |
| --- | --- | --- | --- | --- | --- | --- |
|  | Data (n=101) | Patients completing 6 month FU(N=60) | LTFU (N=20) | p-value^1^ | Non-compliance (N=14) | p-value^2^ |
| Male sex (N, %) | 101 | 18 (30.0) | 8 (38.1) | 0.49 | 7 (50.0) | 0.21 |
| Age (median, IQR) | 101 | 38 (32-48) | 38 (30-48) | 0.69 | 34 (23-46) | 0.14 |
| BMI (kg/m²) | 96 | 21.0 (18.7-23.8) | 20.9 (17.4-26.7) | 0.91 | 20.6 (19.4-22.5) | 0.88 |
| WHO stage (n,%) | 101 |  |  | 0.49 |  | 0.85 |
| 1 |  | 25 (41.7) | 8 (38.1) |  | 5 (35.7) |  |
| 2 |  | 13 (21.7) | 1 (4.8) |  | 4 (28.6) |  |
| 3 |  | 11 (18.3) | 9 (42.9) |  | 4 (28.6) |  |
| 4 |  | 11 (18.3) | 3 (14.3) |  | 1 (7.1) |  |
| **Co-infections** | 101 | 17 (28.3) | 7 (33.3) | 0.78 | 2 (14.3) | 0.5 |
| *TB* | 101 | 12 (20.0) | 5 (23.8) | 0.76 | 0 (0) | 0.11 |
| *Herpes zoster* | 101 | 1 (1.7) | 0 (0) | ND | 2 (14.3) | 0.21 |
| *Kaposi’s sarcoma* | 101 | 0 (0) | 1 (4.8) | 0.53 | 0 (0) | ND |
| *Candidiasis or dermatomycosis* | 101 | 5 (8.3) | 1 (4.8) | 0.53 | 0 (0) | ND |
| *Hepatitis B* | 97 | 2 (3.3) | 0 (0) | 1 | 1 (7.1) | 0.49 |
| *Cryptococcosis* | 93 | 0 (0) | 1 (4.8) | 0.25 | 1 (7.1) | 0.19 |
| *HTLV-1* | 101 | 0 (0) | 1 (4.8) | 0.26 | 3 (21.4) | 0.006 |
|  |  |  |  |  |  |  |
| **Laboratory diagnostics** |  |  |  |  |  |  |
| *Hemoglobin (g/dL)* | 97 | 10.2 (8.3-12.5) | 10.0 (9.0-11.0) | 0.74 | 10.2 (9.1-13.4) | 0.66 |
| *White cell count (*10e9/L)* | 96 | 4.1 (3.4-4.7) | 3.7 (2.6-5.1) | 0.35 | 5.1 (3.0-5.7) | 0.41 |
| *Neutrophils (*10e9/L)* | 71 | 1.77 (1.29-2.46) | 1.76 (1.10-2.45) | 0.64 | 1.39 (1.24-2.17) | 0.31 |
| *Lymphocytes (*10e9/L)* | 76 | 1.29 (1.01-1.69) | 1.26 (0.81-1.67) | 0.76 | 1.86 (0.94-2.14) | 0.29 |
| *Monocytes (*10e9/L)* | 76 | 0.46 (0.37-0.66) | 0.50 (0.23-0.84) | 0.81 | 0.50 (0.41-0.99) | 0.56 |
| *Eosinophils (*10e9/L)* | 83 | 0.19 (0.08-0.57) | 0.20 (0.05-0.47) | 0.48 | 0.18 (0.09-0.86) | 0.94 |
| *Eosinophilia (n,%)* | 63 | 11 (30.6) | 3 (23.1) | 0.73 | 3 (21.4) | 0.65 |
| *Platelets (*10e9/L)* | 96 | 200 (155-296) | 226 (190-270) | 0.6 | 243 (170-318) | 0.38 |
| *HIV viral load (log copies/mL)* | 84 | 4.81 (4.35-5.30) | 4.77 (4.32-5.30) | 0.97 | 5.15 (4.39-5.39) | 0.41 |
| *CD4 count (cells/µL)* | 101 | 185 (67-256) | 155 (51-287) | 0.86 | 134 (95-237) | 0.79 |
| *CD8 count (cells/µL)* | 92 | 801 (538-1265) | 647 (495-1117) | 0.36 | 840 (612-1269) | 0.88 |
| *Creatinine (mmol/L)* | 96 | 60 (48-74) | 56 (46-64) | 0.24 | 59 (44-82) | 0.75 |

Patient characteristics for patients who completed 6-month follow-up; patients who were lost to follow-up (LTFU) and patients who reported non-compliance during the study follow-up.

Body Mass index (BMI); World Health Organization (WHO); Tuberculosis (TB); Human T-cell Lymphotropic Virus-1 (HTLV-1).

^1^ Patients who were LTFU versus those who completed follow-up

^2^ Patients who reported non-compliance versus those who completed follow-up
